# Evaluation of the knowledge of and attitudes towards pharmacovigilance among healthcare students in China: a cross-sectional study

**DOI:** 10.1186/s12909-024-05561-5

**Published:** 2024-05-24

**Authors:** Yan Zhao, Lei Yang, Ruijie Tan, Jing Yuan

**Affiliations:** 1 Center for Drug Reevaluation, NMPA/NMPA Key Laboratory for Research and Evaluation of Pharmacovigilance, Beijing, 100022 P. R. China; 2 Strategic Support Force Characteristic Medical Center, Beijing, 100101 P. R. China; 3 School of Pharmacy, Fudan University, 826 Zhangheng Road, Pudong Dist, Shanghai, 201203 P. R. China

**Keywords:** Pharmacovigilance, Survey, Education, Knowledge

## Abstract

**Background:**

Knowledge of pharmacovigilance (PV) and adverse drug reactions (ADRs) are the core competencies that healthcare students should acquire during their studies. The objective of this study was to assess attitudes towards and knowledge of PV and ADRs among healthcare students in China.

**Methods:**

An online, cross-sectional survey was conducted nationally among healthcare students in China from April through October 2023. Knowledge of PV and ADRs was assessed using a questionnaire based on current PV guidelines. We performed logistic regression analysis to determine the potential factors related to knowledge of and attitudes towards PV and ADRs.

**Results:**

A total of 345 students were included in the analysis. Among the healthcare students who participated in the survey, 225 (65.22%) students correctly defined PV, while only 68 (19.71%) had a correct understanding of ADRs. Among all respondents included in the analysis, only 71 (20.58%) reported having taken a PV course. Pharmacy students were more likely to have taken PV courses at a university and to demonstrate superior knowledge compared to other healthcare students. The logistic regression model revealed that the significant predictors of a higher level of PV knowledge were being female (odds ratio [OR]: 1.76; 95% confidence interval (CI): 1.06–2.92; P value: 0.028) and having previously taken PV-related courses (OR: 2.00; 95% CI: 1.06–3.80; P value: 0.034).

**Conclusions:**

This study revealed that healthcare students’ knowledge of PV and ADRs is unsatisfactory. However, there were a limited number of universities providing PV education. Given the vital role of healthcare professionals in identifying and reporting ADRs, our findings raise significant concerns. Hence, more efforts should be made to enhance PV education for future healthcare professionals.

**Supplementary Information:**

The online version contains supplementary material available at 10.1186/s12909-024-05561-5.

## Background

Pharmacovigilance (PV)—practices related to detecting, monitoring, understanding, and preventing adverse events [1]—is crucial for ensuring drug safety [2]. Although adverse drug events (ADEs) can potentially be detected in premarketing clinical trials, there are noticeable limitations, including narrow patient selection criteria, small sample sizes, and short follow-up periods, which make it nearly impossible to identify all events associated with a drug, particularly rare adverse drug reactions (ADRs) [3, 4]. In fact, rare (1 in 1000) and very rare (1 in 10,000) ADRs are more likely to be reported after the obtainment of market authorization [5]. Hence, strengthening postmarketing PV activities, including the spontaneous reporting of suspected ADRs, is necessary. Spontaneous reporting systems—systems in which suspected ADRs are voluntarily reported by health professionals and pharmaceutical manufacturers [6]—are considered one of the most effective approaches for collecting safety data in the postmarketing phase [7]. However, spontaneous ADE reporting is compromised by underreporting of events. It is estimated that only 2-10% of ADRs are voluntarily reported [8, 9], which further threatens drug safety management in healthcare systems. Thus, it is necessary to design interventions for improving ADR reporting in each country.

Since the 1990s, spontaneous reporting activities to monitor drug safety have been performed in China, including the establishment of the National Adverse Drug Reaction Monitoring System by the National Medical Products Administration (NMPA). Recently, an increasing number of innovative drugs have been granted conditional approval, which generally requires more stringent postmarketing safety surveillance [10]. Consequently, PV has received greater public attention in China and worldwide [11]. The newly revised Drug Administration Law of the People’s Republic of China [12], implemented in December 2019, explicitly proposes establishing a PV system. In 2021, the NMPA released the Guidelines on Good PV Practices [13], which further clarify the provisions needed for safety surveillance activities, such as the reporting, monitoring, risk identification, risk assessment, and control of ADRs.

ADRs represent a significant public health concern, contributing to increased morbidity, mortality, and economic burdens [14]. The reporting of events is heavily reliant on the level of knowledge, professional obligation, and attitude and motivation of healthcare professionals; hence, knowledge of PV and ADR reporting is vital to ensure postmarketing safety. Therefore, healthcare professionals should gain knowledge and develop attitudes towards PV during their undergraduate and graduate studies to develop competence in identifying and reporting ADRs later in their practice. The knowledge of healthcare professionals has been evaluated in many countries [15–18]; however, the knowledge of healthcare students, who may become doctors, pharmacists, and nurses in the future, is limited [19–21], particularly in China. We assessed healthcare students’ attitudes towards and knowledge of PV and ADRs and the potential predictors by conducting a questionnaire survey. We also examined healthcare students’ perceived need for PV courses to be provided for healthcare-related professions or disciplines to optimize the curriculum in the future.

## Methods

### Study design

We used a cross-sectional, questionnaire-based survey design. Medical students were invited to participate in this nationwide survey on knowledge of and attitudes towards PV in China. The survey period was from April 27, 2023, to October 15, 2023.

This study was approved by the Shanghai Ethics Committee for Clinical Research. The requirement for written informed consent from participants was waived by the Shanghai Ethics Committee for Clinical Research. Although written informed consent was not needed, the first page of the survey included an informed consent statement describing that participation was voluntary and anonymous. After completing the questionnaire, the respondents agreed (consented) to participate in the anonymous survey.

### Survey questionnaire development

The questionnaire, which evaluated medical students’ knowledge of and attitudes towards PV, was generated based on references and expert opinions [19–21]. To ensure the quality of the questionnaire, a group of researchers with diverse research backgrounds, including regulatory agency (Y.Z.), medical education (L.Y., J.Y.), clinical practice (L.Y., J.Y.), and industry (J.Y.) backgrounds, reviewed the questionnaire for accuracy and clarity. We also consulted with other researchers. All the feedback from the researchers was considered in the revision of the questionnaire. The questionnaire was then pilot tested among a purposive sample of healthcare students, including five undergraduate students and five graduate students who were majoring in pharmacy, medicine, and traditional Chinese medicine (TCM), to ensure the appropriateness of the questionnaire. The questionnaire was further refined based on the students’ input.

The questionnaire includes four parts. The first part collected demographic and school information, including age, sex, study program, and type of institution; the second part collected information about the respondents’ participation in PV courses; the third part collected information on the respondents’ knowledge about PV-related activities; and the fourth part collected information about the students’ perceived need for PV courses. The questionnaire consisted of single-choice questions, multiple-choice questions, and free text entries. The answers to the single-choice and multiple-choice questions were based on existing guidelines or textbooks. The questionnaire is shown in Appendix.

### Participant recruitment

The research participants included undergraduate and graduate students from medical universities or colleges of comprehensive universities in China. To obtain a more representative sample of students, 10 teaching faculty members or counsellors were chosen as the initial deliverers of the survey. As described in previous studies, the students invited their classmates to participate in the survey via WeChat [22, 23]. WeChat, China’s largest social media platform, has been widely used to conduct online surveys [22]. The institutions of higher education involved in this study included Tsinghua University, Shanghai Medical College of Fudan University, Tongji Medical College of Huazhong University of Science and Technology, Xiangya Medical College of Central South University, and Capital Medical University, which are ranked in the top 20 Best Global Universities for Clinical Medicine in China according to U.S. News [24]. Both Western medicine and TCM courses are offered for healthcare professional students in China [25]. In addition, TCM drugs are regulated by the NMPA and should also meet the PV requirements [26]. Therefore, the survey participants also included students majoring in TCM.

### Questionnaire administration

The respondents received requests to complete the questionnaire via WeChat, including a link to the web-based questionnaire via the internet survey portal (https://www.wjx.cn/). The questionnaires were completed and collected online. To avoid multiple responses from the same student, each WeChat account was allowed to complete the questionnaire only once. A questionnaire was considered valid if (1) all questions were answered; (2) the respondent was majoring in pharmacy, western medicine, TCM, public health, nursing, medical laboratory technology, biomedical engineering, or rehabilitation therapy; and (3) the respondent was still pursuing his or her undergraduate or graduate education. A total of 400 students were invited to participate. 362 students responded to the questionnaire, and the response rate was 80.44%.

### Statistical analysis

We performed descriptive data analysis for each variable. Continuous variables are presented as the mean ± standard deviation if normally distributed; they are expressed as medians and quartiles if not normally distributed. We compared the differences between the means of the two groups using Student’s t-test. Categorical variables are presented as numbers and percentages and were compared using the chi-square test or Fisher’s exact test. To examine the associations between knowledge scores and demographic data, we also used a logistic regression model to estimate odds ratios (ORs) with 95% confidence intervals (CIs). The data were analysed using SAS 9.4. A P value < 0.05 was considered to indicate statistical significance.

## Results

### Characteristics of survey participants

Three hundred sixty-two students completed the questionnaire, and 17 were excluded from the analysis because of missing data or not majoring in healthcare-related disciplines. Hence, a total of 345 students were included in the final analysis. Table 1 shows the characteristics of the students who participated in the survey. Most participants were female (*n* =246; 71.30%), with an average age of 23.38 ± 3.32 years. A total of 46.67% of the participants (*n* = 161) studied at medical universities. Among the 345 survey participants, the majority majored in Western medicine (*n* = 187; 54.20%), followed by pharmacy (*n* = 77; 22.32%) and TCM (*n* = 48; 13.91%). Among the respondents, 175 (50.72%) were undergraduate students, 170 (49.28%) were graduate students.

**Table 1:** Characteristics of the healthcare students who participated in the survey

Characteristics	Total sample (*n* = 345)	*P* value
**Age**, mean ± standard deviation	23.38 ± 3.32	–
**Sex**, n(%)		**< 0.001**
Male	99 (28.70%)	
Female	246 (71.30%)	
**University Type, n(%)**		**0.788**
Medical university	161 (46.67%)	
Comprehensive University	184 (53.33%)	
**Study Program, n(%)**		**0.216**
Undergraduate	175 (50.72%)	
Graduate	170 (49.28%)	
**Specialization, n(%)**		**< 0.001**
Pharmacy	77 (22.32%)	
TCM	48 (13.91%)	
Western medicine	187 (54.20%)	
Others^*^	33 (9.57%)	

*Abbreviations* ADR: adverse drug reaction; TCM: traditional Chinese medicine

*Other disciplines include public health, nursing, medical laboratory technology, biomedical engineering, rehabilitation therapy and other specialties

### Knowledge about PV and ADR reporting

**Fig. 1:**
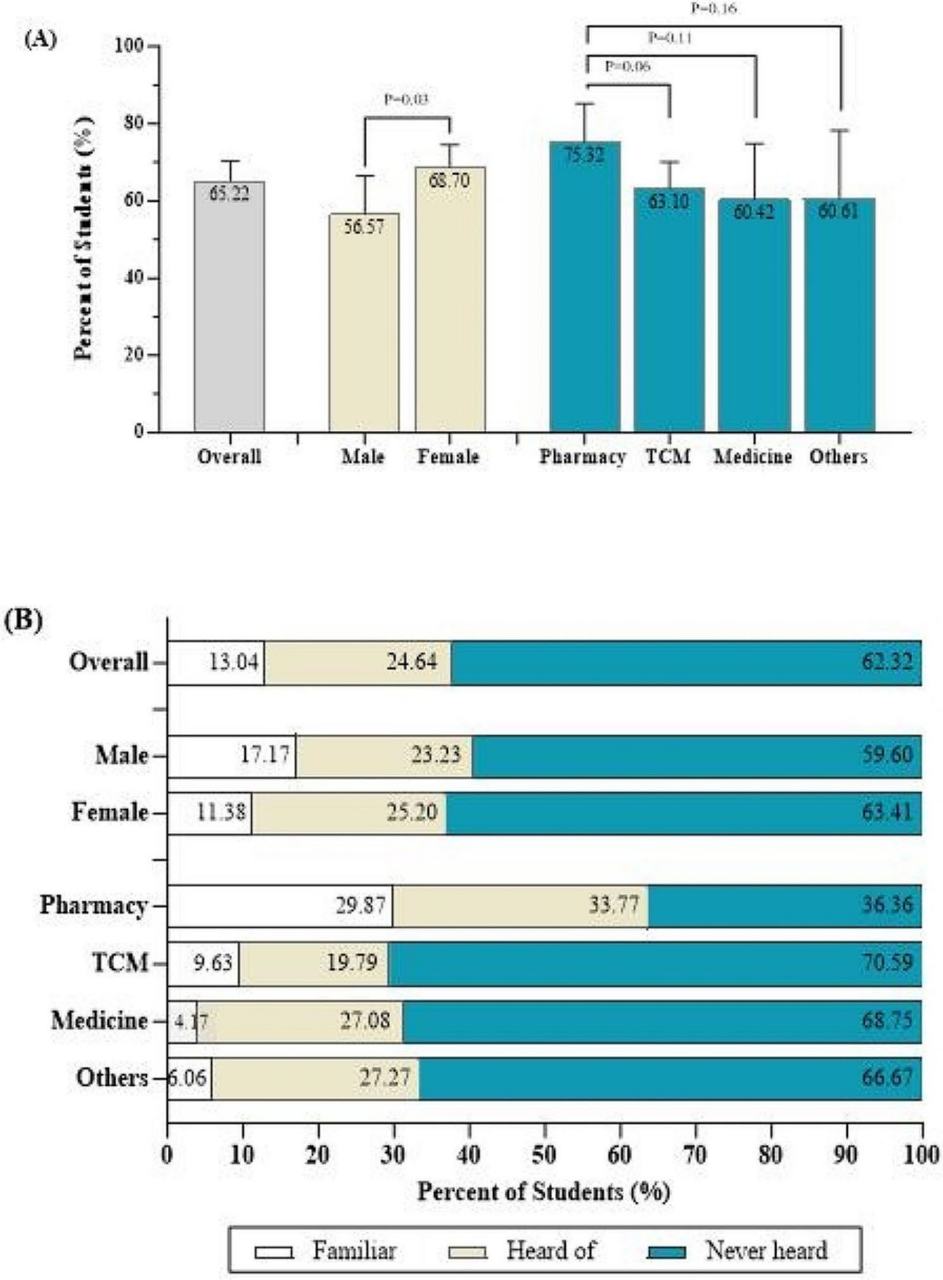
Knowledge of pharmacovigilance among healthcare students in China. (**a**) Proportion of students (%) with a correct understanding of pharmacovigilance by sex and study major. (**b**) The perceived knowledge of pharmacovigilance among healthcare students by sex and study major. TCM: traditional Chinese medicine. *Other disciplines include public health, nursing, medical laboratory technology, biomedical engineering, rehabilitation therapy and other specialties

Among the students included in the analysis, 225 (65.22%) students correctly defined PV (Fig. 1). Female students were more likely to answer correctly than male students were (68.70% vs. 56.57%; *P* = 0.03). The percentage of students who answered correctly was highest among students who majored in pharmacy (75.32%), followed by those who majored in TCM (63.10%), Western medicine (60.42%), and other disciplines (60.61%). Regarding the perceived knowledge of PV, only 45 out of the 345 students (13.04%) thought they were familiar with the PV requirements. The proportion of students who felt that they were familiar with the PV requirements was greater in the male group than in the female group (17.17% vs. 11.38%; *P* = 0.35). The percentage of students who felt that they were familiar with the PV requirements was greater among students majoring in pharmacy (29.87%) than among those with other majors.

**Fig. 2:**
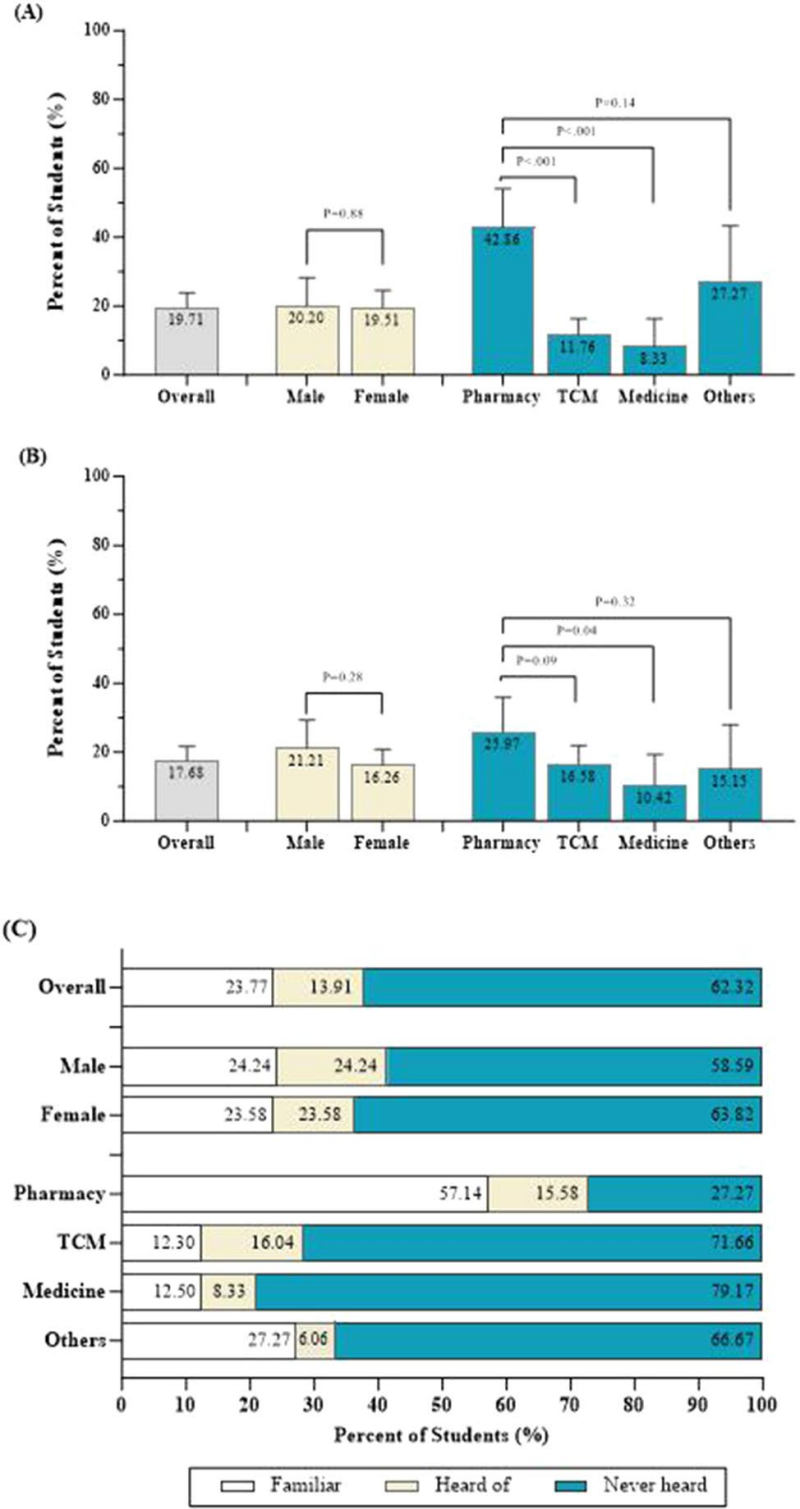
Knowledge of ADR reporting among healthcare students. (**a**) Proportion of students (%) with a correct understanding of ADRs by sex and study major. (**b**) Proportion of students (%) with a correct understanding of ADR reporting time by sex and study major. (**c**) The perceived knowledge of ADRs among healthcare students by sex and study major. TCM: traditional Chinese medicine. *Other disciplines include public health, nursing, medical laboratory technology, biomedical engineering, rehabilitation therapy and other specialties

For the definition of ADRs, 68 (19.71%) students answered correctly (Fig. 2). Students who majored in pharmacy were more likely to define ADRs accurately (42.86%) than were students who majored in TCM (11.76%), Western medicine (8.33%), and other fields (27.27%). Only 61 students (17.68%) knew the correct reporting time for ADRs. The proportion of students who selected the correct answer was highest among students who majored in pharmacy (25.97%), followed by those who majored in TCM (16.58%), Western medicine (10.42%), and other disciplines (15.15%). Among the students who participated in the survey, only 82 out of 345 (23.77%) felt that they were familiar with the requirements of ADR reporting (Fig. 2). The percentage of students who felt that they were familiar with ADR reporting was greater among the group of pharmacy students (57.14%) than among the groups of students who majored in TCM (12.30%), Western medicine (12.50%), and other disciplines (27.27%).

### PV courses offered at the universities

**Fig. 3:**
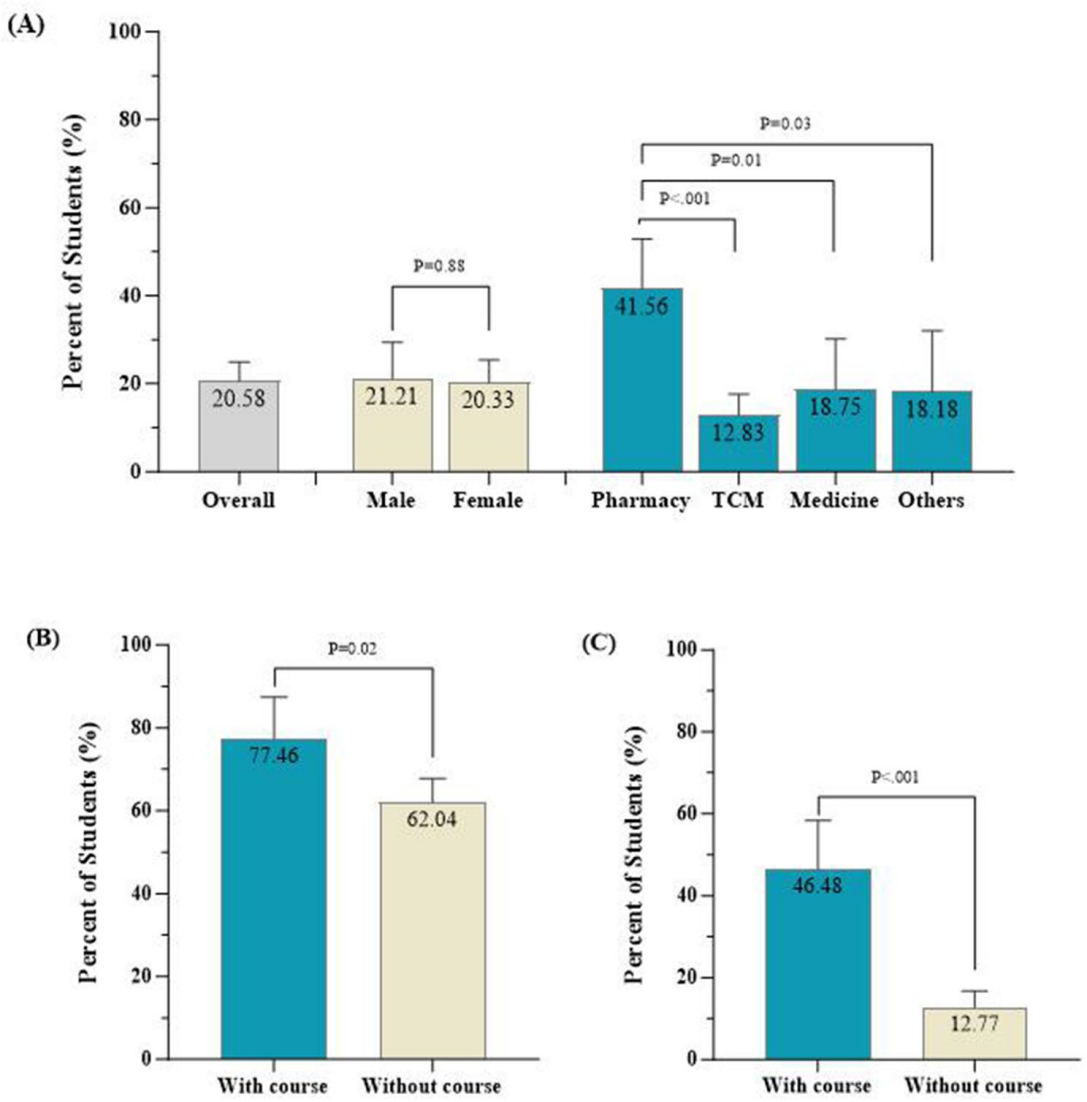
The pharmacovigilance education provided for healthcare students. (**a**) Proportion of students (%) having taken PV courses by sex and study major. (**b**) Proportion of students (%) with a correct understanding of pharmacovigilance by course status. (c) Proportion of students (%) with a correct understanding of ADRs by course status. TCM: traditional Chinese medicine. *Other disciplines include public health, nursing, medical laboratory technology, biomedical engineering, rehabilitation therapy and other specialties

Among all respondents included in the analysis, only 71 (20.58%) reported having participated in a course related to PV (Fig. 3). For PV-related courses, the course curriculum and content should cover the definition and recognition of ADR, PV theory and practices. Pharmacy students were more likely to have taken PV-related courses at a university than students in other healthcare-related professions or disciplines; 41.56% of the pharmacy students had taken PV-related courses, while 12.83% of the students who majored in TCM, 18.75% of the students who majored in Western medicine, and 18.18% of the students who majored in other disciplines had taken PV-related courses. Compared with those who did not take PV-related courses, students who took PV-related courses had better performance in defining PV (77.46 vs. 62.04%; *P* = 0.02) and ADRs (46.48 vs. 12.77%; *P* < 0.001).

**Fig. 4:**
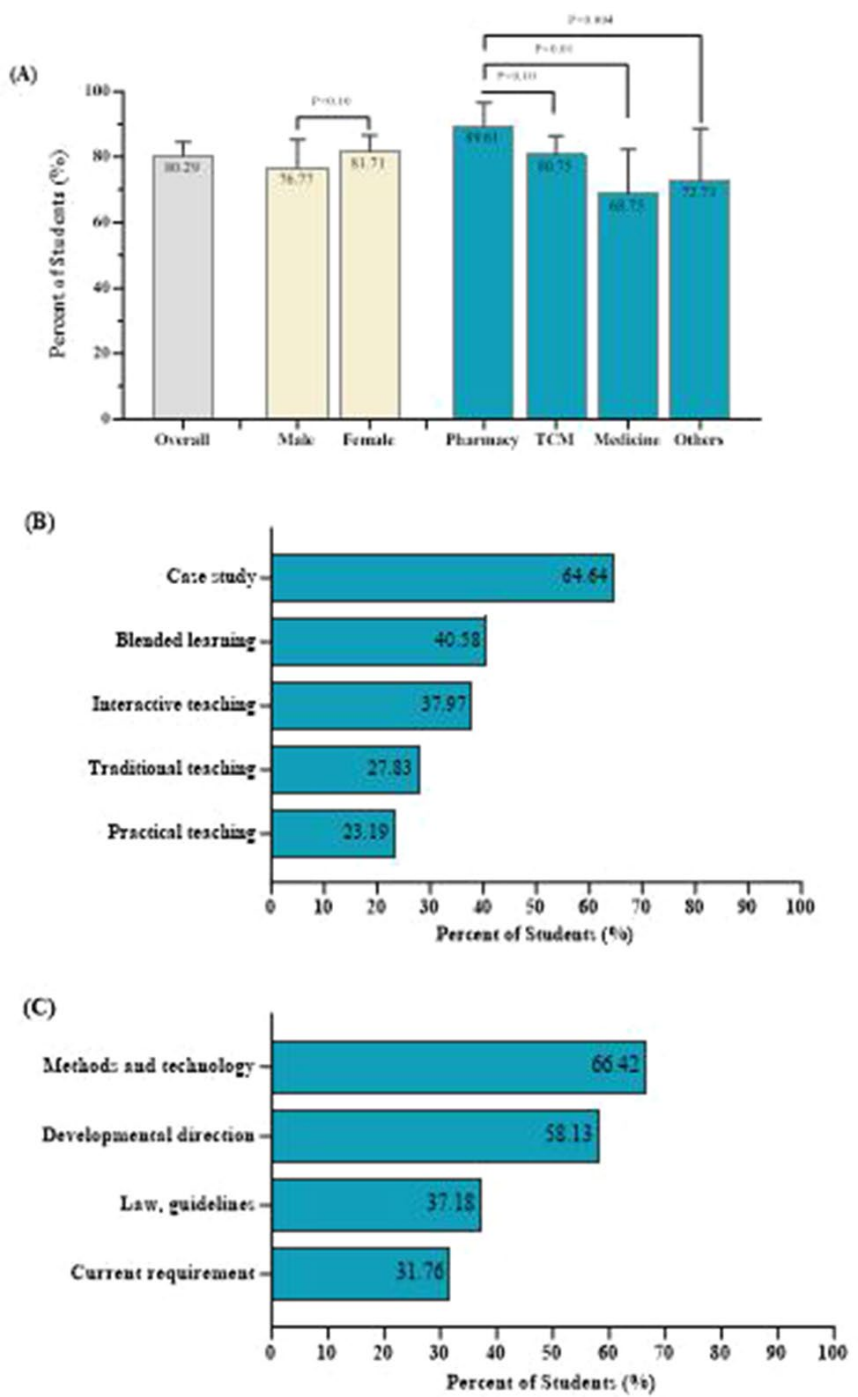
Healthcare students’ perceived needs for a pharmacovigilance course. (**a**) Proportion of students who reported feeling that a PV course was necessary by sex and study major. (**b**) The learning method suggested by healthcare students. (**c**) The course content suggested by healthcare students. TCM: traditional Chinese medicine. *Other disciplines include public health, nursing, medical laboratory technology, biomedical engineering, rehabilitation therapy and other specialties

In terms of the students’ perceived need for a PV-related course, 277 students (80.29%) thought that a PV-related course was necessary for their study (Fig. 4). The percentage of students who felt that PV-related courses were necessary was greater among students majoring in pharmacy (89.61%) than among students majoring in TCM (80.75%), Western medicine (68.75%), and other disciplines (72.73%).

The 277 students who thought a PV-related course was necessary were further asked about their preference for teaching methods and course content (Fig. 4). The most preferred teaching methods were case studies (64.64%), followed by blended learning (40.58%), interactive teaching (37.97%), traditional teaching (27.83%), and practical teaching (23.19%). In terms of course content, the students wanted the course to cover recent progress in methods and technology (66.42%), followed by developmental direction in PV (58.13%), PV-related laws and guidelines (37.18%), and current requirements (31.76%).

### Factors influencing knowledge of PV and ADRs

Table 2 shows the associations between the healthcare students’ characteristics and their overall knowledge of PV and ADR reporting. Multivariate logistic regression analysis revealed that significant predictors of a higher level of PV knowledge were being female (OR: 1.76; 95% CI: 1.06–2.92; P value: 0.028) and having previously taken a PV-related course (OR: 2.00; 95% CI: 1.06–3.80; P value: 0.034). For the definition of ADRs, graduate students had better knowledge than did undergraduate students (OR: 2.47; 95% CI: 0.99–6.12; P value: 0.052). Students who majored in pharmacy were more likely to correctly define ADRs than those who majored in TCM (OR: 0.21; 95% CI: 0.09–0.46; P value: 0.034), Western medicine (OR: 0.14; 95% CI: 0.03–0.57; P value: 0.048), or other healthcare-related professions or disciplines (OR: 0.73; 95% CI: 0.27–1.93; P value: 0.094).

**Table 2:** Factors associated with knowledge of pharmacovigilance and ADRs

Characteristics	PV		ADR	
	OR (95% CI)	*P* value	OR (95% CI)	*P* value
**Age**	0.97 (0.87–1.08)	0.542	0.90 (0.79–1.04)	0.163
**Sex**				
Male				
Female	1.76 (1.06–2.92)	0.028	1.02 (0.52–2.00)	0.952
**University Type**				
Medical university				
Comprehensive University	1.30 (0.68–2.50)	0.428	0.86 (0.39–1.92)	0.712
**Study Program**				
Undergraduate				
Graduate	0.81 (0.37–1.77)	0.598	2.47 (0.99–6.12)	0.052
**Specialization**				
Pharmacy				
TCM	0.81 (0.40–1.61)	0.524	0.21 (0.09–0.46)	0.034
Western medicine	0.54 (0.18–1.58)	0.451	0.14 (0.03–0.57)	0.048
Others^*^	0.57 (0.23–1.41)	0.507	0.73 (0.27–1.93)	0.094
**Having previously taken PV-related courses**	2.00 (1.06–3.80)	0.034	4.85 (2.55–9.23)	< 0.001
**Feels that PV courses are necessary**	0.89 (0.49–1.62)	0.706	0.74 (0.33–1.64)	0.453

*Abbreviations* ADR: adverse drug reaction; TCM: traditional Chinese medicine; PV: pharmacovigilance

*Other disciplines include public health, nursing, medical laboratory technology, biomedical engineering, rehabilitation therapy and other specialties

## Discussion

According to this extensive survey of PV knowledge and perceived needs among healthcare students, two-thirds of the students could correctly define PV. Nevertheless, fewer than 20% of the students correctly defined ADRs and the correct ADR reporting time. Our survey also revealed that only 1 in every five survey respondents had taken PV-related courses at their university, which partially explained the unsatisfactory level of PV knowledge among healthcare students in China. In other countries, more than half of healthcare students could correctly define PV or ADRs [17, 27], which was higher than the proportion of students in China. PV was recently developed in China and has not been incorporated into the curriculum for healthcare professionals or disciplines. Our findings underscore the importance of enhancing PV education for healthcare students.

Overall, fewer than 20% of the healthcare students knew about ADRs, while this proportion was greater than 50% among pharmacy students, possibly because of the variability of the curriculum dedicated to PV. This finding is also consistent with the literature [28, 29]; pharmacists tend to have greater knowledge of the definition of ADRs than other healthcare professionals, potentially due to their specialized pharmaceutical training. Similarly, in this survey, pharmacy students had better knowledge of ADRs than did other healthcare students, potentially because they had taken pharmacy administration courses, which cover the regulation requirements for PV and ADRs. In China, a pharmacy administration course is an elective course required for a pharmacy curriculum. Most of the top pharmacy universities include a pharmacy administration course in their curriculum, offered to undergraduate students in their 3rd or 4th year and graduate students in their 1st year. However, pharmacy administration courses or other courses covering PV and ADRs are not generally offered for other healthcare students, potentially causing a knowledge gap in the field of PV. Therefore, there is a need for greater PV education for healthcare students and continuous education for healthcare professionals.

In addition to the differences in the curricula of the study programs, the observed gap in PV knowledge may also contribute to the difference in attitudes towards PV between pharmacy students and other healthcare students. According to our analysis, the proportion of students who recognized the importance of PV was greater among students majoring in pharmacy than among students majoring in other healthcare disciplines [30], which is consistent with the literature. In other countries, it has been reported that pharmacy students have more significant attitudes and perceptions towards PV than other healthcare students [21]. Given that most healthcare students fail to correctly define ADRs, there is considerable doubt about the ability of future healthcare professionals (e.g., doctors and pharmacists) to report ADRs in a timely manner [30]. Therefore, offering more PV courses for healthcare professionals is warranted to increase awareness of PV and ADRs.

Our findings indicate an urgent need to promote PV education for healthcare students. Our analysis revealed that the students had limited knowledge in defining and reporting ADRs. Hence, it is necessary to include PV education in the curricula of healthcare programs. In terms of perceived needs, the respondents expressed an urgent desire to take PV-related courses. In particular, healthcare students suggested covering case studies by incorporating interactive teaching methods. They also expressed their willingness to learn new analytical skills. The need for PV education stands in sharp contrast to the fact that few universities in China offer PV-related courses. In addition to changing the curricula of study programs, which might take longer to implement, students could be offered an online course on PV education. The majority of the students expressed interest in e-learning. For example, the online course provided by the Uppsala Monitoring Centre could be used as a teaching module to develop courses in China [31]. Online courses could also be used in continuous education for healthcare professionals [32].

With the increased number of new drugs with postmarketing safety requirements, the recent trend of pharmaceutical regulation is to “chase the high line” to encourage innovation and development. At the same time, “guarding the bottom line” ensures the safety of medicines [11]. In this new era, the NMPA aims to achieve a rapid-response surveillance system involving governments, industries, healthcare professionals, and the public; thus, improving knowledge about PV is highly important. To date, Chinese healthcare professionals have a relatively limited knowledge of defining and reporting ADRs [33], potentially contributing to underreporting in China. Hence, more efforts should be directed at enhancing PV education.

Several limitations of this study should be considered. First, we developed the questionnaire based on PV guidelines in China. Although we included an expert panel and a group of students to pilot the survey questionnaire, the instrument has not been validated. Furthermore, these questions only represent students’ understanding of the definitions of PV and ADRs; hence, students’ knowledge of PV may be inadequately reflected in this analysis. Third, despite our efforts to recruit healthcare students from universities across China [24], the respondents accounted for only a small proportion of healthcare students in China. Hence, our findings may not be generalizable to students from other universities. Finally, selection bias may exist because students who were confident in their knowledge of PV were more likely to participate in the survey. As such, we cannot exclude the possibility that students with greater knowledge of PV were included. Most of the students who participated in the survey were from top medical universities in China, and the knowledge of PV and ADRs of students from other universities might be even worse.

## Conclusions

This study revealed that healthcare students’ knowledge of PV and ADRs is relatively low. However, a limited number of universities provide PV education. This is of great concern given the vital role of healthcare professionals in identifying and reporting ADRs. Hence, we call for strengthening PV education for future healthcare professionals at both the undergraduate and graduate levels.

### Electronic supplementary material

Below is the link to the electronic supplementary material.

Supplementary Material 1

## Data Availability

The datasets used and/or analysed during the present study are available from the corresponding author upon reasonable request.

## Abbreviations

ADR: Adverse drug reaction
CI: Confidence interval
NMPA: National Medical Products Administration
OR: Odds ratio
PV: Pharmacovigilance
TCM: Traditional Chinese medicine
