# Supplementary material for: Evaluation of the knowledge of and attitudes towards pharmacovigilance among healthcare students in China: a cross-sectional study
Source: BMC Med Educ. 2024 May 24;24:570. doi: 10.1186/s12909-024-05561-5 (PMC11127336; doi:10.1186/s12909-024-05561-5)
Supplement: Supplementary file 1 — Supplementary Material 1 [file 12909_2024_5561_MOESM1_ESM.docx]

Survey for Pharmacovigilance knowledge

Part I. Demographics

1. Gender

①Male ②Female

2. Age (numerical value)

3. Current Study Stage

①freshman

②sophomore

③junior

④senior

⑤fifth year

⑥Master student

⑦Bachelor-straight-to-doctorate student

⑧Doctoral student

4. Your current school is a

①Comprehensive University

②Medical University

③Other ____

5. What is your major ?

①Pharmacy

②Traditional Chinese Pharmacy

③Preventive Medicine and Public Health

④Clinical Medicine

⑤Basic Medicine

⑥Dental Medicine

⑦Traditional Chinese Medicine

⑧integrated traditional and Western medicine

⑨Nursing

⑩others____

Part II. Participation in PV courses

6. Have you ever participated in courses related to "pharmacovigilance" or "monitoring of adverse drug reactions"?

①Yes ②No

7. (If you choose ① for question 6) Where did you take the course?

①In school ②MOOC ③Other ____

8. (If you choose ① for question 6) When did you take the course?

①Undergraduate ②Master's ③Other ____

9. (If you choose ① for question 6) Is the course compulsory or elective ____?

①compulsory ②elective ③other ____

Part III. Knowledge about PV-related activities

10. Do you understand the concept of pharmacovigilance?

①Yes ②No

11. What is the definition of pharmacovigilance?

①Pharmacovigilance refers to the discovery of the pattern of drug damage, so as to reduce and eliminate drug damage, to ensure the safety of medication

②Pharmacovigilance is the scientific research and activities related to the discovery, evaluation, understanding and prevention of adverse reactions or any other problems that may be related to drugs

③pharmacovigilance is the study of drug safety

④Pharmacovigilance is the evaluation of the risk-benefit ratio of medication

12. Do you know the meaning of ADR/ADE in pharmacovigilance?

①Clearly know ②Probably know ③Heard of it ④Don't know/haven't heard of it

13. Do you know what ADR in pharmacovigilance means?

①ADR is any adverse event that occurs in a patient or a subject of a clinical study of a drug

②ADR is an unrelated or unexpected harmful reaction to a qualified drug that occurs when the drug is used in its normal dosage

③ADR refers to the use of the same drug in the process, in a relatively concentrated period of time, the region, to a certain number of people's physical health or life safety damage or threat to the event that requires emergency treatment

④ADR refers to a suspected or unanticipated serious adverse reaction whose clinical manifestations are of a nature and severity that exceeds the information already available in the investigator's manual of the test drug, the instruction manual of the marketed drug, or the summary of the product characteristics

14. Do you know which of the following situations need to be reported?

①Adverse drug reactions reported in the literature, but it is not certain whether it is the holder's product

②Adverse drug reactions reported in the literature, and the suspected drug is the holder's product

③Serious adverse reactions occurring outside of China

④All of the above need to be reported

15. Do you understand the requirements of pharmacovigilance in China?

①very well ②generally ③have heard of it ④not at all

16. China requires that new and serious adverse drug reactions should be reported ____, deaths should be reported ____, and other adverse drug reactions should be reported ____.

①within 10 days, immediately, within 30 days ②within 10 days, within 3 days, within 30 days

③within 15 days, immediately, within 30 days ④within 15 days, within 3 days, within 60 days

Part IV. Perceived need for PV courses

17. Are you interested in pharmacovigilance course?

①Yes ②No ③I don't care

18. Do you think there is a need for a pharmacovigilance course?

①Yes ②No ③Doesn't matter

19. (If you choose ① for question 18) If you attend a pharmacovigilance course, what are your reasons for attending? (Multiple choices allowed)

①Needed for corporate employment

②Needed for hospital employment

③Needed for scientific research

④Just interested

⑤To get enough credits

⑥Other ____

20. (If you choose ② or ③ for question 18) The reasons why you don't take the pharmacovigilance course are (You can choose more than one. List in order of priority)

①Already taken similar courses

②Credits already meet graduation requirements

③May conflict with other courses

④Busy with labs/internships/job hunting/graduate school exams

⑤Not interested

⑥Too tired

⑦Other ____

21. (If you choose ① for question 18) What kind of teaching style do you want? (You can choose more than one)

①Group discussion and interactive teaching

②Actual case study

③Traditional lecture

④Offline lecture

⑤Online and offline synchronous classroom

22. (If you choose ① for question 18) In addition to the main content of the course, what content would you like to be introduced? (You can choose more than one. List in order of priority)

①New technology of pharmacovigilance

②Direction of development of pharmacovigilance discipline

③Current situation and trend of foreign pharmacovigilance development

④Case study of Chinese pharmacovigilance related laws and regulations

⑤Others ____

23. What other expectations and suggestions do you have for the course?
